# Supplementary material for: Implementation outcomes of the waves for change community-based task-shared prevention intervention for adolescent mental health in South Africa
Source: Glob Ment Health (Camb). 2025 Jun 30;12:e77. doi: 10.1017/gmh.2025.10033 (PMC12322778; doi:10.1017/gmh.2025.10033)
Supplement: Davies et al. supplementary material [file S2054425125100332sup001.docx]

## List of Supplementary Documents

[Supplementary Document 1: Waves for Change programme details 1](#_Toc199257006)

[Supplementary Document 2: Further details on adolescent focus group discussions 4](#_Toc199257007)

[Supplementary Document 3: Site assessment questionnaire 5](#_Toc199257008)

[Supplementary Document 4: Evidence of adoption of the 5 pillars by coaches 7](#_Toc199257009)

[Supplementary Document 5: Additional quotations 9](#_Toc199257010)

[Supplementary Document 6: Responses to the COREQ checklist 11](#_Toc199257011)

## Supplementary Document 1: Waves for Change programme details

W4C works with an excess of 2500 children annually, aged between 10 and 16 years old. Participants are referred from a network of quintile 1-3 schools, clinics, hospitals and other community-based organisations in the most under-served communities surrounding five ocean-side sites in South Africa. Criteria for referral include if children have experienced interpersonal trauma, have few support structures, exhibit behavioural aggression and reactivity, and/or deterioration in self-care.

Coaches aged 18-25 are employed in the programme, from the same communities as the participants. Most have their senior certificate (Grade 12), but do not have any other formal qualifications before entering the organisation. They undergo a values-based recruitment process, police checks, and need to demonstrate an interest in sport mentoring and/or supporting youth development.

The W4C programme uses the 5 Pillar Method to introduce and strengthen protective factors for children exposed to or experiencing toxic stress, and improve their ability to self-regulate while living in adverse conditions. The programme is made up of comprehensive coach training and a curriculum for the participants. These two arms are interdependent and play equal roles in the attainment of outcomes in the participants. The Method was primarily designed to optimise the natural benefits of fun, group-based sport, recreation and play, to move distressed, pre-symptomatic adolescents towards wellness.

**Coach Training**

The first phase of training is five full days, and focuses on the key theories underlying the 5 Pillar Method, which is informed by the implications of toxic stress and adversity on child and adolescent development and mental health. The training orients coaches to techniques that create a sense of safety, trust, belonging and attachment in a trauma-informed manner.

The second phase of training is conducted concurrently with the children’s programme throughout the year. The training is staggered to allow for gradual learning and continuous personal and professional development for the coaches. Training is held weekly, and involves teaching the curriculum for the following week. At these sessions, coaches practice and enact all the activities they will teach so that they fully understand them and are able to confidently teach them to the children. During this training, coaches also practice embedding the 5 protective factors (identified in the 5 Pillar Method) using a simple empathic coaching skills (e.g. praise and listening), and through structuring surfing in a way that builds confidence. Coaches use a simple coaching routine (energise, check-in, self-regulation based activity from scaffolded curriculum, surfing / play time, check out).

Coaches are supervised through weekly group and individual check-ins, and regular programme fidelity assessments are conducted. They also attend weekly mental health and wellbeing debriefs with a registered mental health professional (such as a mental health professional or registered counsellor) and are referred for further care if necessary. Coaches are given child protection training to support the formation of healthy boundaries with children. Child protection concerns raised by coaches are addressed within 24 hours by a Child Protection team that follows protocols for the disclosures (e.g. rape - referral to Thuthuzela Care Centres). In this way, coaches are required to ‘contain’ participants with traumatic disclosures but not to counsel or address themselves.

In addition, they receive first aid training and weekly surf training to increase their competence in surfing. Coaches are also employed for a minimum of two years, and opportunities are provided where possible to move upward in the organisation as site managers or trainers. Following their employment at W4C, they are mentored to transition into further work through a local mentoring NGO, ‘SAYes’ (https://sayesmentoring.org/).

**Curriculum**

The five pillars are integrated into a curriculum that offers a time-bound set of activities designed to improve social connectedness and self-regulation skills. It follows three broad phases of ‘building strong connections, strong minds, and a strong sense of future’, following an implementation manual. The time frame follows the four South African school terms, over a period of 10 months. Each weekly session is repeated twice to reinforce learnings and behaviour change. The curriculum is available publicly from the organisation.

The first phase of the curriculum focuses on building healthy relationships through encouraging trust, empathy, respect and communication, and creating a shared culture of protection between coaches and peers. The second phase focuses on developing skills to cope with stress by mastering deep breathing, meditation, and a deeper understanding of strengths, and the third phase involves preparing to be independent by identifying how and where children will use the learned skills in day-to-day life.

After 10 months of weekly engagement, participants graduate to an aftercare pathway which includes weekend ‘surf clubs’ as a drop-in service which they can attend until the age of 17. Following this, they are supported to transition to new opportunities. Some participants apply to become coaches themselves.

**Implementation of the model**

Programme sessions are run for different groups every week day, and ‘surf club’ sessions on Saturdays. As each group comes weekly, approximately 10 sessions are held by coaches every week at each of the five sites. Sessions are two hours long, and can cater for up to 60 children at a time, but with a maximum coach to child ratio of 1:7.

Each session involves a welcome, changing into wetsuits, doing energisers, doing check-ins, a ‘take-5’ breathing technique, discussing the theme of the day related to social connectedness and self-regulation, doing a fun, water-based activity and/or surfing, a group debrief/checkout, and receiving a meal. Children do not require any beach or swimming skills/experience to participate fully. Transport is provided by the organisation from schools or community pick-up points and children are always supervised by a coach.

Self-regulation tools are taught through fun games, and catchy phrases such as the ‘Take-5’ (five mindful breaths), the ‘power hand’ (identifying strengths), identifying their ‘boiling points’ (anger thresholds), using ‘snap claps’ to demonstrate appreciation and praise of others, and having a ‘banana culture’ of ‘respect protect, communicate and share’, which is encapsulated in an easy hand gesture.

**Website**: <https://waves-for-change.org/>

## Supplementary Document 2: Further details on adolescent focus group discussions

**Inclusion of adolescents in the Focus Group Discussions**

It was decided that adolescents would partake in FGDs and not individual interviews so that more adolescents could be included, and that there was adequate representation of the three different programme sites and of attendance levels amongst them.

Interviews were conducted in English, which was participants’ first or second language. There were two occasions when adolescent participants asked for clarification of a question in isiXhosa, in which case fellow participants did so for them. The researcher could also speak basic isiXhosa and ensured that these were being translated correctly.

Adolescents in each FGD were part of the same surf group and demonstrated a strong degree of cohesion in their groups. It therefore appeared that they were comfortable sharing their experiences in front of their peers, although this could not be guaranteed.

## Supplementary Document 3: Site assessment questionnaire

| Timestamp |
| --- |
| Your Name |
| Site |
| Date of Assessment |
| Number of children present |
| Number of coaches present |
| What type of session did you observe? |
| Please mark whether the following was done: [Was a beach assessment form completed ] |
| Please mark whether the following was done: [Was a daily plan in place] |
| Please mark whether the following was done: [Was the site manager present for the session] |
| Please mark whether the following was done: [Children were greeted enthusiastically] |
| Please mark whether the following was done: [App attendance register was taken of all children present] |
| Please mark whether the following was done: [Children were supervised while changing into wetsuits] |
| Please mark whether the following was done: [Children were supervised by coaches when walking to the beach] |
| Please note any comments/concerns about today's pre-session planning: |
| Were there enough wetsuits for the participants |
| Were there enough boards for the participants |
| Please note any comments/concerns about today's equipment and resources |
| Please mark whether the following was done: [Coaches/Children participated with energy in the energiser/warm-up activity] |
| Who led the energiser/warm up |
| Which COACH led the energiser/warm-up activity? Please include the coach's names (not the child's name!) |
| Please note any highlights/concerns about today's energiser/warm-up activity (e.g. did children lead an energiser/warm-up activity) |
| Please mark whether the following was done: [Kids and coaches sit in a safe circle and practice calm breathing] |
| Please mark whether the following was done: [Coaches do a check-in of last week's Teachable Moment] |
| Did coaches ask children 'what' they learnt last week and 'how' and 'when' they used it? (Yes/No?) Please give examples of children's feedback: |
| Who Led the take 5/breathing activity |
| Which coach led the Take 5/breathing activity? Please include their names |
| Please note any challenges/highlights about today's Take 5/breathing activity: |
| Please mark whether the following was done: [Did the coaches explain the lesson/behaviour we want to teach for this week?] |
| Please mark whether the following was done: [Did children engage in this week's lesson/behaviour activity?] |
| Please mark whether the following was done: [Did coaches engage in this week's lesson/behaviour activity?] |
| Please mark whether the following was done: [Did the coaches do a debrief summary/check-in with the children AFTER this week's lesson/activity?] |
| During the debrief with children, did coaches ask children 'what' they learnt today and 'how' and 'when' they used it today or can use it in their lives? (Yes/No?) Please give examples of children's feedback: |
| Please note any highlights/challenges about today's Teachable Moment/lesson: |
| Please mark whether the following was done: [Did coaches encourage female participant to surf?] |
| Please mark whether the following was done: [Did Female participants surf/tried to surf?] |
| Please mark whether the following was done: [Did Female coaches take surfboards and go to the backline with children?] |
| Please mark whether the following was done: [Did Male coaches stay with children who didn't want to surf?] |
| Please note any comments/concerns about today's Free Time(e.g. did all children go into the water) |
| Please mark whether the following was done: [Did the coaches prep the additional tools designed for this session, BEFORE the session started?] |
| Please mark whether the following was done: [Did the coaches use/engage with the additional tools?] |
| Please mark whether the following was done: [Did the children use/engage with the additional tools?] |
| Please mark whether the following was done: [Did the teachers/external staff assist with the children (e.g. help with changing, help with difficult behaviour)] |
| Please note any highlights/concerns/challenges with today's specialised group: |
| Please mark whether the following was done: [Children were supervised walking back to the site from the beach/while changing out of their wetsuits] |
| Please mark whether the following was done: [Did male coaches help prepare/serve food?] |
| Please mark whether the following was done: [Coaches did a debrief on today's session, after children left] |
| Was the session, in any way, disrupted? |
| If the session was disrupted, what did you to fix it? |
| Please note any highlights/concerns with today's session closing (e.g. wetsuit washed and hung up, food was served, children ate safely) |
| What session was the assessment completed on? |

## Supplementary Document 4: Evidence of adoption of the 5 pillars by coaches

Coaches and adolescents provided accounts of the coaches’ utilisation of the 5-Pillars. These participants provided frequent descriptions of coaches adopting the behaviours and teachings required from the Method, for four of the five pillars, and moderately, for the fifth pillar.

Coaches demonstrated being consistent and caring adults (Pillar 1) through describing how they do regular check-ins with adolescents, show interest in their lives, and provide care and support where needed. *“We look out for the kids, ask them how they’re feeling, we do check ins with the kids, like okay, how are you? How are things at home? How was your day at school and stuff? To show each other that we care for one another” (C2).*

In doing so they create an emotionally safe space for them (Pillar 2): *“The space we make for them is, we celebrate everything for them, so if they do a nice thing, we celebrate it. So we have like a ‘banana culture’ which is the values of ‘respect, protect, communicate and share’. So what we teach them those values and we see them actually like practicing that and they're respecting each other” (C11).*

Adolescents endorsed the creation of a safe space through statements such as: “*I think they are kind, I think they are protective of us, like even when you do bad things and they ask you and you tell them, they are not like judgemental or anything like that*” (AD16).

The coaches demonstrated implementation of the activities and exercises from the protocol with energy, fun and enthusiasm (Pillar 3). A coach explained that *“if you do the energiser and are not energetic the kids won’t participate... You have to give them the best energy so that they can feel free during that time” (C5).*

All participants provided examples of coaches adopting and teaching the self-regulation and coping skills that they had been given in the curriculum until that point (Pillar 4)*.* Adolescents explained that *“They teach us ‘take fives’ and snap claps always, and play games to keep us calm and not sad” (AD22),* and*, “they teach us how to keep ourselves calm when we're angry or sad. [They do this by] giving us ‘take fives’ and teaching us about ‘banana cultures’” (AD19).*

**Uptake by adolescents**

Uptake and utilisation of the 5-Pillars by adolescents was also demonstrated in the data. A coach explained that *“what we teach them, those values, we see them actually practicing that, and they're respecting each other”* (C11). In receiving care and praise from caring adults, children learn to *“do the same toward others”* (SK3), thus forming caring connections and meaningful relationships with others. There were also reports of adolescents’ regulating negative behaviour outside of the programme, leaving negative friendships, learning social and emotional skills, changing behaviour patterns, and having improved awareness of emotions and behaviour, in their schools. For example, a teacher said that at school, the W4C adolescents encourage each other to conduct themselves appropriately and point out each other’s negative behaviours, demonstrating practicability and suitability of their learnings: *“Even one of the learners said, ‘No man don’t do that, we are W4C, our coach said we mustn’t do this’!”* (SK5).

## Supplementary Document 5: Additional quotations

**Appropriateness**

Q1:

“Through exposure to these spaces where the participants are learning to relax, it starts to open them up to different ways with regulating and alternative ways of behaving I suppose. It’s the hope that they can get positively reinforced by the coaches, and it’s also important that we don’t take too much credit, I think a lot of children and youth know what positive pro-social behaviours are. They can be quite instinctual but the environments that they are often in don’t necessarily allow for them. Whereas, if you're at the beach and you have fun and with people that you trust and relax. Those things, come naturally, right? “It’s not Like it’s because suddenly Waves for Change has arrived that those participants learn those things... it’s just that it’s a space where they feel comfortable to do it and then coaches observe, celebrate, reinforce.” (S1)

Q2:

“So, if you're in a space where your parasympathetic system gets to do its magic for a little bit and you can relax for a little while, what that means for your body’s functioning. If you're in a space where you have access to a caring adult and what it does for your self-esteem. If you're in a space where you get to learn new things that make you feel good about yourself, all of the kind of impact that has on the internal world of the participant and their thoughts and their feelings and also their physical functioning. That then, hopefully goes back with them into their community or into their future. So, I believe that the 5-Pillar approach, within the capacity of Waves for Change, within the context that we're working, is most likely to have an impact on that internal world of our participants that they then take back into their home environments. I think talk therapy can be incredibly powerful; I think there’s all sorts of different …I'm not saying those things don’t work, but for our participants, I think we have, no, I *know*, that we have incredible impact on their self-concept, on their ability to regulate, on their sense of hope and future. And that then goes with them.” (S1)

Q3:

A staff member explained that “the HRV study informed our learnings around dosage and how long it was taking our participants to settle into the space… One of our takeaways was that it’s taking on average about eight weeks for our participants to, for their systems to settle, and be impacted by the positive environment that we’re creating through the five pillars… [So we] extended our curriculum and added a new first phase that focuses entirely on coach behaviours to create safe spaces and build strong connections. And only then do they start teaching the participants new behaviours and skills. …if your system hasn’t settled yet and you're not feeling comfortable and safe and relaxed in the space, you're going to be much less interested with learning” (S1).

**Adoption**

Q4:

“And then there’s the very structured curriculum that teach them things like, breathing activities and there’s lots of discussions around, you know, we don’t just demonstrate and teach the breathing activity, we have discussions with the children that encourage, that are kind of informed by behaviour activation. So, encouraging self‑awareness within the participants about how that behaviour made them think and feel.

And then planning together or identifying together how you can use those. So, if you’ve done a take‑five breathing at the beach, recognising that helps you feel calm and focused or whatever it might be. So, when else in your life can you use this when you want to feel that way. For example, Pair surfing - helping my friend made me feel really good about myself. I recognise that I was actually really great at listening and so I'm going to use listening to connect with my mom or whatever it might be. So, that’s kind of a third aspect of the training part where I guess it’s these practising behaviours and then the conversations that build awareness around how does this make us think and feel and other ways that we could use them. So it’s actually an agency thing.

Interviewer: And that’s a really, really important part of the whole programme/ curriculum, is actually reflecting back on what they’ve done. So, that everything is transferable, right? So, whether it’s the skill or the behaviour or even just the experience. Like, what did you love about today and helping. You know you feel that way at home or in your community. So, it’s really important that we’re not just teaching things, it’s important that the participants recognise their agency and their self‑reliance and ability to use those things themselves.” (S1)

**Acceptability**

Q5:

*“The space we make for them is, we celebrate everything for them, so if they do a nice thing, we celebrate it. So we have like a ‘banana culture’ which is the values of ‘respect, protect, communicate and share’. So what we teach them those values and we see them actually like practicing that and they're respecting each other” (C11).*

**Fidelity**

Q6:

“It’s an interesting one because I think, we haven’t always got the balance right around independence and adaptation within the coaches. That’s something that we’ve tried to encourage, while at the same time, the core ingredients have to remain. Like the method has to remain and... where that line is, for interpretation, We’ve sometimes left it a bit too open and that is then actually not empowering and enabling for coaches to do their job. And at the end of the day we are also asking them to do a job and a service.

Participant: But at other times, we’ve been way too prescriptive, which and has not allowed for their local understanding and their own essential knowledge to come through. So, and that’s been part of the curriculum iterations that I mentioned is, that there’s been times where they were like, 200 page documents because of us providing so much information.

Participant: And then, there’s been times where it’s been almost too much the other end and not enough and that’s been disempowering for coaches. So, how we train it and encourage it, as an underlying culture, we’ve had to learn.” (S1)

## Supplementary Document 6: Responses to the COREQ checklist

**Consolidated criteria for reporting qualitative studies (COREQ): 32-item checklist**

**Domain 1: Research team and reflexivity**

**Personal Characteristics**

1. Interviewer/facilitator Which author/s conducted the interview or focus group? – *TD*

2. Credentials What were the researcher’s credentials? E.g. PhD, MD – *PhD, Registered Counsellor*

3. Occupation What was their occupation at the time of the study? – *Researcher*

4. Gender Was the researcher male or female? - *Female*

5. Experience and training What experience or training did the researcher have? – *15 years of training and experience in conducting in-depth closed- and open-ended interviews and focus groups with participants ranging in age, race, gender, profession and mental health status.*

**Relationship with participants**

6. Relationship established Was a relationship established prior to study commencement? – *The researcher was introduced to participants through W4C contacts.*

7. Participant knowledge of the interviewer. What did the participants know about the researcher? e.g. personal goals, reasons for doing the research. – *The researcher explained reasons for doing the research, and professional position, but did not discuss personal goals or details.*

8. Interviewer characteristics What characteristics were reported about the interviewer/facilitator? e.g. Bias, assumptions, reasons and interests in the research topic. *The following was reported: All interviews were conducted by the primary researcher (TD), who is independent to the organisation, has extensive individual and group interviewing experience, and is also trained as a Registered Counsellor with the Health Professions Council of South Africa. Coaches and adolescents were assured that their feedback would be confidential and that what they shared would not impact their participation or employment in any way. Social desirability bias was minimised through TD being independent to W4C and not known to the participants (apart from two staff members).*

**Domain 2: Study design**

**Theoretical framework**

9. Methodological orientation and Theory. What methodological orientation was stated to underpin the study? e.g. grounded theory, discourse analysis, ethnography, phenomenology, content analysis.

*The study was underpinned by a qualitative content analysis approach, using thematic analysis (Braun & Clarke, 2006) as the analytical method.*

**Participant selection**

10. Sampling How were participants selected? e.g. purposive, convenience, consecutive, snowball. *Adult participants were purposively selected for their roles with or in W4C. Adolescent participants were from six groups who attended the weekly W4C programme at the three programme sites in Cape Town (two groups per site). A de-identified list of all adolescents attending the three programme sites was obtained from W4C and initially stratified by site and gender. Following this, the adolescents were randomly selected using a random number generation form.*

11. Method of approach How were participants approached? e.g. face-to-face, telephone, mail, email. *– participants were approached via email, telephone and face-to-face.*

12. Sample size How many participants were in the study? - *69*

13. Non-participation How many people refused to participate or dropped out? Reasons? – *Nine out of 48 adolescent participants did not arrive on the various days of their respective group interviews, but no reasons were given.*

**Setting**

14. Setting of data collection Where was the data collected? e.g. home, clinic, workplace. *– Online, and at the organisation.*

15. Presence of non-participants Was anyone else present besides the participants and researchers? – *No.*

16. Description of sample What are the important characteristics of the sample? e.g. demographic data, date. – *Characteristics of the adolescent sample are described in Table 1. Professional occupations of the adult participants are listed in Table 3.*

**Data collection**

17. Interview guide Were questions, prompts, guides provided by the authors? Was it pilot tested? – *The primary researcher developed the topic guides and sent them to the fellow authors for comment. Guides were not piloted but were adjusted if necessary following the first interview.*

18. Repeat interviews Were repeat interviews carried out? If yes, how many? – *Yes, multiple interviews and focus groups were conducted, reported in the methods section.*

19. Audio/visual recording Did the research use audio or visual recording to collect the data? – *Yes, every interview was audio recorded.*

20. Field notes Were field notes made during and/or after the interview or focus group? – *yes, the primary researcher made field notes during and after the interviews and focus groups.*

21. Duration What was the duration of the interviews or focus group? – *interviews took between 30-60 minutes and focus groups took between 60-90 minutes.*

22. Data saturation Was data saturation discussed? – *No, a set number of participants was decided upon before implementation.*

23. Transcripts returned Were transcripts returned to participants for comment and/or correction? *No these were not returned to participants, however there were multiple groups of participants interviewed to increase validity.*

**Domain 3: analysis and findings**

**Data analysis**

24. Number of data coders How many data coders coded the data? - *Data was primarily coded by TD. To assess for consistency and accuracy of coding, codes and themes were presented to all study authors halfway through analysis for feedback.*

25. Description of the coding tree Did authors provide a description of the coding tree? - *No*

26. Derivation of themes Were themes identified in advance or derived from the data? – *Both a-priori and derivative themes were used.*

27. Software What software, if applicable, was used to manage the data? – *Nvivo 12 was used to analyse the data.*

28. Participant checking Did participants provide feedback on the findings? – *No*

**Reporting**

29. Quotations presented Were participant quotations presented to illustrate the themes / findings?

Was each quotation identified? e.g. participant number. *– Yes*

30. Data and findings consistent Was there consistency between the data presented and the findings? - *Yes*

31. Clarity of major themes Were major themes clearly presented in the findings? - *Yes*

32. Clarity of minor themes Is there a description of diverse cases or discussion of minor themes? – *Yes.*
